# Preoperative evaluation of MRI features and inflammatory biomarkers in predicting microvascular invasion of combined hepatocellular cholangiocarcinoma

**DOI:** 10.1007/s00261-023-04130-6

**Published:** 2023-12-19

**Authors:** Juan Zhang, Wei Dong, Wanmin Liu, Jiazhao Fu, Tian Liao, Yinqiao Li, Lei Huo, Ningyang Jia

**Affiliations:** 1 Department of Radiology, Eastern Hepatobiliary Surgery Hospital, Third Affiliated Hospital of Naval Medical University, Shanghai, China; 2 Department of Pathology, Eastern Hepatobiliary Surgery Hospital, Third Affiliated Hospital of Naval Medical University, Shanghai, China; 3 Department of Radiology, Tongji Hospital, School of Medicine, Tongji University, Shanghai, China; 4 Department of Organ Transplantation, Changhai Hospital, First Affiliated Hospital of Naval Medical University, Shanghai, China; 5 Department of Ultrasound, Changsha Hospital of Traditional Chinese Medicine, Changsha, China; 6 School of Health Science and Engineering, University of Shanghai for Science and Technology, Shanghai, China

**Keywords:** Combined hepatocellular cholangiocarcinoma, Magnetic resonance imaging, Microvascular invasion, Inflammatory biomarkers, Risk factors

## Abstract

**Purpose:**

Microvascular invasion (MVI) is a significant prognostic factor in combined hepatocellular cholangiocarcinoma (cHCC-CCA). However, its diagnosis relies on postoperative histopathologic analysis. This study aims to identify preoperative inflammatory biomarkers and MR-imaging features that can predict MVI in cHCC-CCA.

**Methods:**

This retrospective study enrolled 119 patients with histopathologically confirmed cHCC-CCA between January 2016 and December 2021. Two radiologists, unaware of the clinical data, independently reviewed all MR image features. Univariable and multivariable analyses were performed to determine the independent predictors for MVI among inflammatory biomarkers and MRI characteristics. The area under the receiver operating characteristic (ROC) curve (AUC) was used to evaluate the diagnostic performance.

**Results:**

Multivariable logistic regression analysis identified four variables significantly associated with MVI (*p* < 0.05), including two inflammatory biomarkers [albumin-to-alkaline phosphatase ratio (AAPR) and aspartate aminotransferase-to-neutrophil ratio index (ANRI)] and two MRI features (non-smooth tumor margin and arterial phase peritumoral enhancement). A combined model for predicting MVI was constructed based on these four variables, with an AUC of 0.802 (95% CI 0.719–0.870). The diagnostic efficiency of the combined model was higher than that of the imaging model.

**Conclusion:**

Inflammatory biomarkers and MRI features could be potential predictors for MVI in cHCC-CCA. The combined model, derived from inflammatory biomarkers and MRI features, showed good performance in preoperatively predicting MVI in cHCC-CCA patients.

**Graphical abstract:**

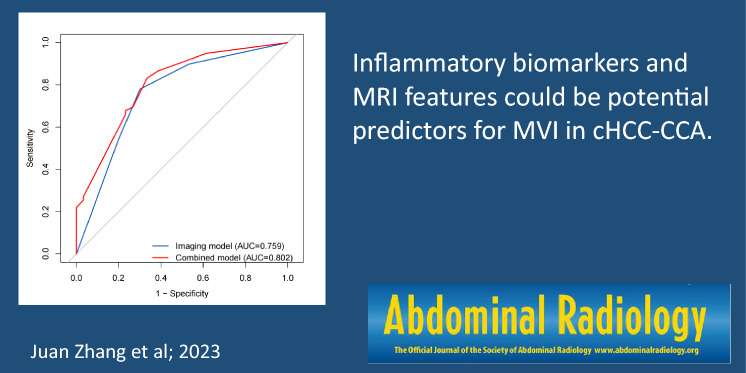

**Supplementary Information:**

The online version contains supplementary material available at 10.1007/s00261-023-04130-6.

## Introduction

Combined hepatocellular cholangiocarcinoma (cHCC-CCA) is a rare primary liver carcinoma (PLC) that exhibits both hepatocytic and cholangiocyte differentiation [1]. Its prevalence in PLCs ranges from 0.4 to 14.2% [2]. Some reports suggest that cHCC-CCA has a poorer survival rate than hepatocellular carcinoma (HCC) and intrahepatic cholangiocarcinoma (ICC) [1, 3, 4], while others indicate that its prognosis is similar to ICC but worse than HCC [5]. Surgery is the primary treatment for most patients with resectable cHCC-CCA. However, the recurrence rate of cHCC-CCA after surgical resection can reach 80% within 5 years, and the 5-year survival rate is lower than 30% [6–8].

Microvascular invasion (MVI) is a significant prognostic factor in cHCC-CCA, associated with early recurrence and low survival rates [9–14]. For liver cancers with MVI, the marginal range of surgical resection should be expanded, and additional postoperative adjuvant therapy should be considered due to MVI’s indication of more aggressive tumor behavior [15, 16]. Early and accurate prediction of MVI can benefit treatment decision making and prognosis evaluation in cHCC-CCA patients. However, MVI is a histological manifestation that requires postoperative pathological examination.

Currently, MRI features are more commonly used in the preoperative diagnosis of MVI in HCC [17] and ICC [18]. However, due to its low prevalence, little is known about the preoperative application of MRI features to predict MVI in cHCC-CCA patients. Inflammation, which promotes tumor cell proliferation, invasion, and angiogenesis, has gained considerable attention for its influence on tumor development and metastasis [19]. Inflammatory biomarkers are used to assess the response to systemic inflammation. Some inflammatory biomarkers have been proven as independent risk factors for MVI in HCC [20–25], but their relationship with MVI in cHCC-CCA patients is not yet fully understood. This study aims to explore the preoperative predictive value and role of MRI features and inflammatory biomarkers for MVI in cHCC-CCA patients.

## Materials and methods

### Study population

**Fig. 1:**
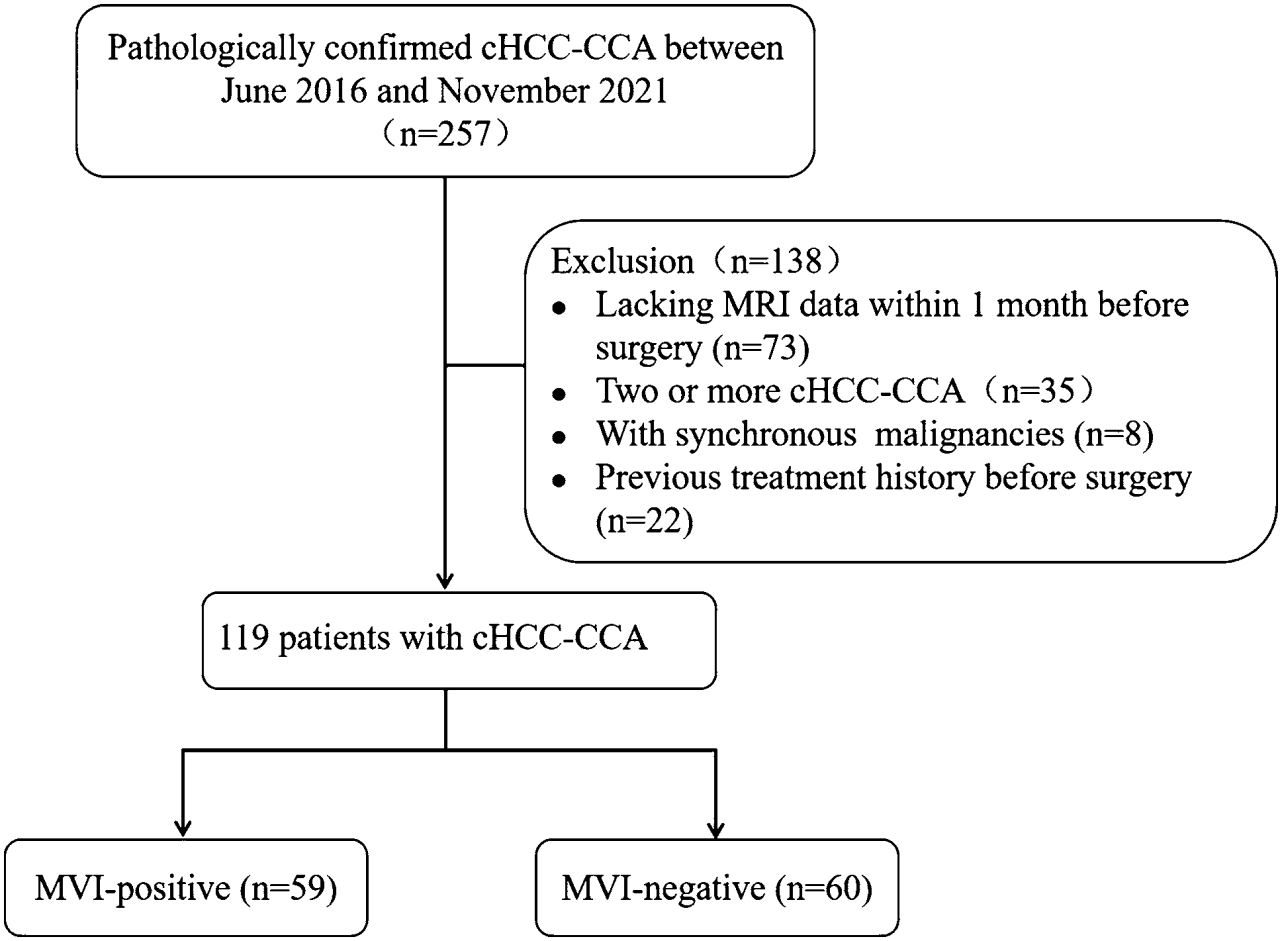
Flowchart of this study population

This retrospective study was approved by the Ethics Committee of our institution. A total of 257 consecutive patients diagnosed with cHCC-CCA by postoperative pathology between January 2016 and December 2021 were included. Inclusion criteria were (1) a single mass, (2) MRI examination within 30 days before surgery, and (3) no history of any related treatment before surgery. Exclusion criteria were (1) lack of preoperative imaging data, (2) multiple lesions or concurrent other malignancies, such as HCC and ICC, and (3) patients who underwent hepatectomy more than once. Ultimately, 119 patients were enrolled in this study, and all cases were in accordance with the 2019 WHO classification [26]. The flow chart of patient registration is shown in Fig. 1.

### Preoperatove MRI

All MRI examinations were performed using a Signa Infinity Twin Speed 8-channel body coil 1.5 T scanner (GE, USA). Patients fasted for 4 h before the scan. The following sequences were used: transverse T1-weighted breath-hold in-phase and opposed-phase gradient echo sequence, and transverse respiratory-navigated T2-weighted single-shot fast spin-echo sequences. Diffusion-weighted imaging (DWI) was performed using a spin-echo-echo planar imaging (SE-EPI) sequence with *b* values of 0 and 600 s/mm^2^. Gadolinium meglumine (Gd-DTPA, Beilu, China) at a dose of 0.1 mmol/kg was injected into the patient’s median cubital vein at a flow rate of 2.0 mL/s using a high-pressure syringe. Enhanced scans of the arterial, portal, and delayed phases were performed at 22–25 s, 55–65 s, and 120–160 s after Gd-DTPA injection, respectively. The detailed parameters of each acquisition sequence are provided in Table S1.

### Imaging feature analysis

All MRI scans were evaluated independently by two radiologists (Z.J and H.L, with 7 and 14 years of abdominal imaging experience, respectively) using the picture archiving and communication system (PACS; Pathspeed, Pathspeed, GE Medical Systems Integrated Imaging Solutions). Both radiologists were aware that all patients had cHCC-CCA but were blinded to any clinical data or pathological findings. In case of disagreement, further analysis was conducted by both readers, followed by a consensual discussion.

The following imaging features of the lesions were evaluated on the unenhanced scan: (a) tumor shape (globular, lobulated, or irregular); (b) margin (smooth or non-smooth); (c) tumor location (right, left, or other liver lobes); (d) intratumoral hemorrhage; (e) intratumoral fat deposits; (f) intratumoral necrosis; (g) peritumoral bile duct dilation; (h) hepatic capsule retraction; and (i) DWI target sign. Dynamic enhancement features included (A) Arterial phase: (a) non-rim arterial phase hyperenhancement (APHE); (b) peritumoral enhancement; (B) Portal venous phase: (c) non-peripheral washout; (d) enhanced capsule; (C) Delayed phase: (e) delayed central enhancement; (D) Other features: (f) nodule-in-nodule architecture; (g) mosaic architecture. In addition, MRI features were classified according to LI-RADS Version 2018 [27]: LR-TIV (intravenous tumor), LR-M (definitely or probably malignant, not HCC-specific), or LR1-5 (1, definitely benign; 2, probably benign; 3, indeterminate probability of HCC; 4, probably HCC; or 5, definitely HCC).

### Clinical variables and pathology evaluation

The following clinical data were collected from medical records: (a) demographic characteristics, including age and gender; (b) history of hepatitis B; (c) maximum tumor diameter, categorized into groups of 1–2 cm, 2–5 cm, and > 5 cm; (d) liver functional parameters, including alanine aminotransferase (ALT), aspartate aminotransaminase (AST), γ-glutamyltranspeptidase (GGT), albumin (ALB), pro-albumin (pro-ALB), total bilirubin (TB), and direct bilirubin (DB); (e) the following parameters were derived from neutrophil count, lymphocyte count, monocyte count, platelets, and liver function parameters: neutrophil-to-lymphocyte ratio (NLR = N/L), platelet-to-lymphocyte ratio (PLR = P/L), lymphocyte-to-monocyte ratio (LMR = L/M), albumin-to-alkaline phosphatase ratio (AAPR = ALB/ALP), aspartate aminotransaminase-to-platelet ratio (APRI = AST/PLT), aspartate aminotransferase-to-neutrophil ratio index (ANRI = AST/N), (alkaline phosphatase + gamma-glutamyl transpeptidase)/lymphocyte ratio (AGLR = (ALP + GGT)/L), γ-glutamyl transferase-to-platelet ratio index (GPRI = GGT/PLT), neutrophil to pro-albumin ratio index (NRPI = N/Pro-ALB), γ-glutamyl transferase-to-albumin ratio (GAR = GGT/ALB), and γ-glutamyl transferase-to-lymphocyte ratio (GLR = GGT/L); (f) tumor biomarkers, including alpha-fetoprotein (AFP), carcinoembryonic antigen (CEA), carbohydrate antigen 19-9 (CA19-9), and protein induced by vitamin K absence or antagonist-II (PIVKA-II).

The pathological features of hepatectomy patients were evaluated by experienced pathologists, who had at least 10 years of experience in reading tissue sections of liver cases and were unaware of the patients’ MRI characteristics and clinical indicators. Based on the pathological findings, the patients were divided into two groups: those who were positive for microvascular invasion (MVI) and those who were negative for MVI.

### Statistical analysis

Continuous variables that conformed to a normal distribution were expressed as the mean ± standard deviation (SD) and compared using an independent samples *t* test. Non-normally distributed continuous variables were represented as the median (25th, 75th percentile) and compared using the Mann–Whitney *U* test. Categorical variables were reported as frequencies and compared using the *χ*^2^ test. The interobserver agreement between two radiologists for imaging features was evaluated using the Cohen’s Kappa. The optimal cutoff points for NLR, PLR, LMR, AAPR, APRI, ANRI, GPRI, AGLR, NRPI, GAR, and GLR were determined using receiver operating characteristic (ROC) curves. Variables that reached statistical significance in the univariate analysis were included in a multivariate logistic regression analysis to investigate independent risk factors for MVI. A *p* value of less than 0.05 was considered statistically significant. All statistical analyses were performed using SPSS (version 26.0; IBM) and R software (version 3.6.1).

## Results

### Clinicopathological characteristics

A total of 119 patients with combined hepatocellular cholangiocarcinoma (cHCC-CCA) were enrolled in this study, including 94 males (79.0%) and 25 females (21.0%). The comparison of clinicopathologic characteristics of cHCC-CCA patients is summarized in Table 1. Of the patients, 59 (49.6%) were grouped as MVI positive, while 60 (50.4%) were classified as MVI negative. The mean age of the patients was 52.4 ± 10.4 years. The average maximum tumor diameter was 4.2 ± 2.6 cm, and the mean values for NLR, PLR, LMR, AAPR, APRI, ANRI, GPRI, AGLR, NRPI, GAR, and GLR were 1.94, 124.70, 3.51, 0.75, 0.08, 4.63, 256.52, 0.39, 0.03, 0.92, and 28.10, respectively. The clinical data revealed that the serum AFP level was > 400 ng/mL in the MVI-positive group, which was higher than in the MVI-negative group (*p* = 0.020). For the inflammatory biomarkers, patients with higher AAPR (*p* = 0.025) and NRPI (*p* = 0.037) levels, and lower APRI (*p* = 0.014) and ANRI (*p* = 0.013) levels, tended to develop MVI. Other clinical indicators did not differ significantly between the two groups.

**Table 1:** Clinicopathologic characteristics of patients with cHCC-CCA

Clinical parameters	MVI-positive (*n* = 59)	MVI-negative (*n* = 60)	*p* value
Age (years)	54.4 ± 10.5	53.9 ± 10.5	0.245
Gender			0.241
Male	44 (74.6)	50 (83.3)	
Female	15 (25.4)	10 (16.7)	
Largest diameter (cm)			0.261
1–2 cm	8 (13.6)	15 (25)	
2–5 cm	33 (55.9)	31 (51.7)	
≥ 5 cm	18 (30.5)	14 (23.3)	
Hepatitis B virus			0.851
Absent	11 (18.6)	12 (20)	
Present	48 (81.4)	48 (81.4)	
Cirrhosis			0.940
Absent	35 (59.3)	36 (60)	
Present	24 (40.7)	24 (40)	
AFP			0.020*
≤ 400 ng/mL	42 (71.2)	53 (88.3)	
> 400 ng/mL	17 (28.8)	7 (11.7)	
PIVKA-II			0.625
≤ 40	22 (37.3)	25 (41.7)	
> 40	37 (62.7)	35 (58.3)	
CA19-9			0.127
≤ 39 ng/mL	50 (84.7)	44 (73.3)	
> 39 ng/mL	9 (15.3)	16 (26.7)	
CEA			0.319
≤ 10 ng/m	59 (100)	59 (98.3)	
> 10 ng/m	0 (0)	1 (1.7)	
ALT, U/L	26 (18,37)	22 (16,93)	0.713
AST,U/L	27 (20,34)	23 (18,51.3)	0.786
GGT, U/L	44 (28,77)	41.5 (17.3,71.5)	0.282
ALB, g/L	42.3 ± 3.3	43.0 ± 3.6	0.438
Pro-ALB, g/L	206.6 ± 56.1	222.7 ± 58.6	0.334
TiBL, μmol/L	13.5 ± 4.3	15.1 ± 5.7	0.231
DBL, μmol/L	5.1 ± 1.9	5.6 ± 2.7	0.427
NLR			0.235
≤ 1.94	30 (50.8)	24 (40)	
> 1.94	29 (49.2)	36 (60)	
PLR			0.272
≤ 124.70	42 (71.2)	37 (61.7)	
> 124.70	17 (28.8)	23 (38.3)	
LMR			0.074
≤ 3.51	12 (20.3)	21 (35)	
> 3.51	47 (79.7)	39 (65)	
AAPR			0.025*
≤ 0.75	50 (84.7)	58 (96.7)	
> 0.75	9 (15.3)	2 (3.3)	
APRI			0.014*
≤ 0.08	10 (16.9)	2 (3.3)	
> 0.08	49 (83.1)	58 (96.7)	
ANRI			0.013*
≤ 4.63	15 (25.4)	5 (8.3)	
> 4.63	44 (74.6)	55 (91.7)	
AGLR			0.074
≤ 256.52	26 (44.1)	17 (28.3)	
> 256.52	33 (55.9)	43 (71.7)	
GPRI			0.156
≤ 0.39	39 (66.1)	32 (53.3)	
> 0.39	20 (39.9)	28 (46.7)	
NRPI			0.037*
≤ 0.03	46 (78)	55 (91.7)	
> 0.03	13 (22)	5 (8.3)	
GAR			0.082
≤ 0.92	33 (55.9)	24 (40)	
> 0.92	26 (44.1)	36 (60)	
GLR			0.169
≤ 28.10	33 (55.9)	26 (43.3)	
> 28.10	26 (44.1)	34 (56.7)	

Data are presented as the mean ± standard deviation, median (25th, 75th percentile), and the number of patients (%). The *p* value represents the statistical difference between the MVI-positive and MVI-negative groups

*HBV* hepatitis B virus, *AFP* α-fetoprotein, *PIVKA-II* protein induced by vitamin K absence or antagonist-II, *CA 19-9* carbohydrate antigen 19-9, *CEA* carcinoembryonic antigen, *ALT* alanine aminotransferase, *AST* aspartate aminotransferase, *GGT* γ-glutamyltranspeptidase, *ALB* albumin, *Pro-ALB* pro-albumin, *TBL* total bilirubin, *DBL* direct bilirubin, *NLR* neutrophil-to-lymphocyte ratio, *PLR* platelet-to-lymphocyte ratio, *LMR* lymphocyte to monocyte ratio, *AAPR* albumin-to-alkaline phosphatase ratio, *APRI* aspartate aminotransferase to platelet ratio index, *ANRI* aspartate aminotransferase-to-neutrophil ratio index, *AGLR* (alkaline phosphatase + γ-glutamyltranspeptidase) to lymphocyte ratio, *GPRI* γ-glutamyltranspeptidase to platelet ratio index, *NRPI* neutrophil to prealbumin ratio index, *GAR* γ-glutamyltranspeptidase to albumin ratio, *GLR* γ-glutamyltranspeptidase to lymphocyte ratio, *MVI* microvascular invasion

*Indicate statistical significance

### MRI characteristics of cHCC-CCAs

**Fig. 2:**
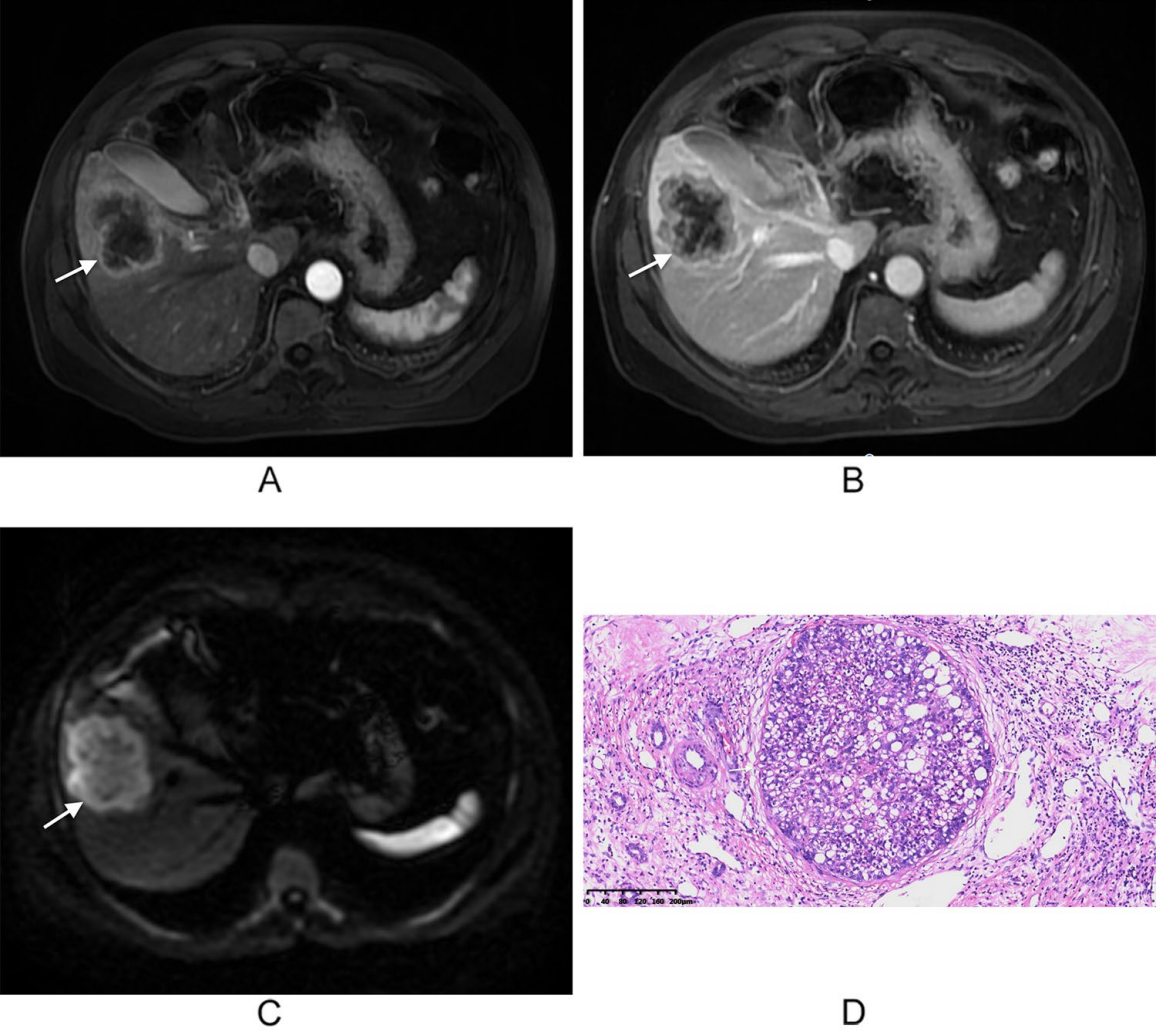
MR images of a 59-year-old man with cHCC-CCA and hepatitis B virus infection, categorized as LR-M with MVI. **A** An arterial phase image shows a lobulated mass with rim enhancement in segment V of the right lobe, accompanied by peritumoral enhancement. **B** The portal venous phase image shows central enhancement of the lesion. **C** A diffusion-weighted image (*b* = 600 s/mm^2^) displays targetoid appearance with peripheral hyperintensity and central relative hypointensity (arrow). **D** A histopathological image of microvascular invasion shows one tumor embolus near the tumor (H&E staining; magnification, ×10)

**Fig. 3:**
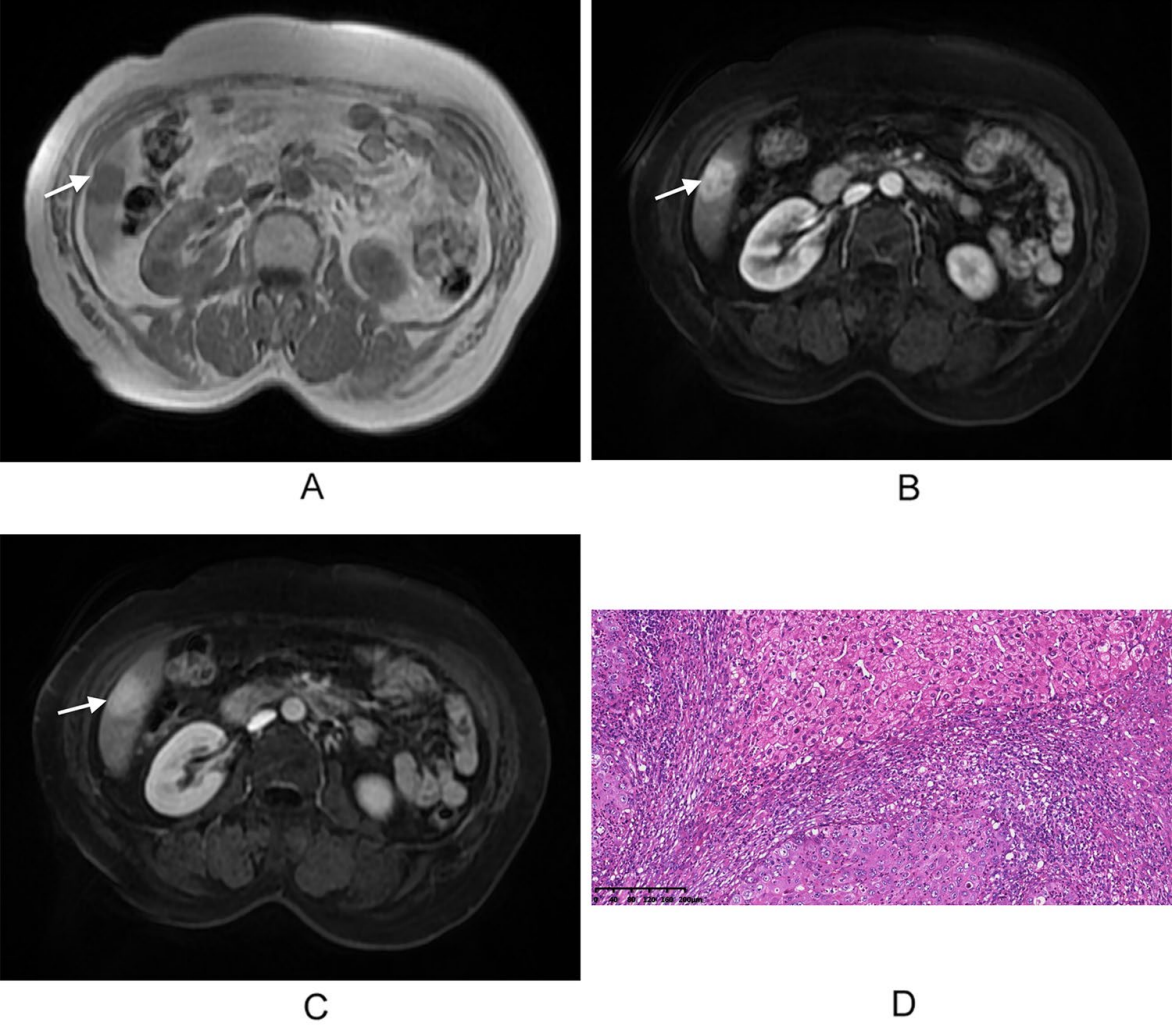
MR images of a 74-year-old woman with cHCC-CCA, without hepatitis virus infection or MVI. **A** A T1WI image shows a nodule in segment VI of the liver, with surface retraction (white arrows). **B** An axial arterial phase image displays a hypervascular nodule (white arrow). **C** A delayed phase image shows persistent enhancement of the lesion. **D** A histopathological image of microvascular invasion shows no tumor embolus (H&E staining; magnification, ×10)

Among the MR-imaging features, arterial phase peritumoral enhancement (78% vs. 30%, *p* < 0.001) and non-smooth margin (66.1% vs. 44.3%, *p* = 0.013) were significantly more frequent in patients with MVI positive compared to those without MVI. Of the 119 patients, 56 were categorized as LR-4/5, 56 as LR-M, and 7 as LR-TIV according to the Liver Imaging Reporting and Data System (LI-RADS) categories. The composition of the final LI-RADS categories did not differ significantly between the two groups (*p* > 0.05). Other characteristics were not significant between the two groups (Table 2). The inter-reader agreements for MR-imaging features were good to excellent (Cohen’s kappa = 0.7608–0.879) (Table S2). Representative images of cHCC-CCA with MVI are displayed in Fig. 2, and images of cases without MVI are displayed in Fig. 3.

**Table 2:** Comparison of MR-imaging features of cHCC-CCA

MRI features	MVI-positive (*n* = 59)	MVI-negative (*n* = 60)	*p* value
Tumor location			0.609
Left liver lobe	13 (22)	13 (21.7)	
Right liver lobe	46 (78)	46 (76.7)	
Other location	0 (0)	1 (1.6)	
Shape			0.672
Globular	28 (47.5)	32 (53.4)	
Lobulated	13 (22)	14 (23.3)	
Irregular	18 (30.5)	14 (23.3)	
Margin			0.013*
Smooth	20 (33.9)	34 (56.7)	
Non-smooth	39 (66.1)	20 (43.3)	
Intratumoral hemorrhage			0.872
Absent	45 (76.3)	45 (75)	
Present	14 (23.7)	15 (25)	
Fat deposition			0.134
Absent	53 (89.8)	58 (96.7)	
Present	6 (10.2)	2 (3.3)	
Intratumoral necrosis			0.313
Absent	32 (54.2)	38 (63.3)	
Present	27 (45.8)	22 (36.7)	
Non-rim APHE			0.296
Absent	38 (64.4)	33 (55)	
Present	21 (35.6)	27 (45)	
Arterial phase peritumoral enhancement			< 0.001*
Absent	13 (22)	42 (70)	
Present	46 (78)	18 (30)	
Washout			0.169
Absent	33 (55.9)	26 (43.3)	
Present	26 (44.1)	34 (56.7)	
Enhancing capsule			0.741
Absent	41 (69.5)	40 (66.7)	
Present	18 (30.5)	20 (33.3)	
Delayed central enhancement			0.404
Absent	36 (61)	41 (68.3)	
Present	23 (23)	19 (31.7)	
Nodule in nodule			0.144
Absent	45 (76.3)	52 (86.7)	
Present	14 (23.7)	8 (13.3)	
Mosaic architecture			0.303
Absent	36 (61)	42 (70)	
Present	23 (39)	18 (30)	
Peritumoral bile duct dilatation			0.522
Absent	52 (88.1)	55 (91.7)	
Present	7 (11.9)	5 (8.3)	
Hepatic capsule retraction			0.795
Absent	41 (69.5)	43 (71.7)	
Present	18 (30.5)	17 (28.3)	
Target sign on DWI			0.053
Absent	46 (78)	37 (61.7)	
Present	13 (22)	23 (38.3)	
LI-RADS categorization			0.258
LR-4/5	24 (40.7)	32 (53.4)	
LR-M	30 (50.8)	26 (43.3)	
LR-TIV	5 (8.5)	2 (3.3)	

The data are presented as the number (%) of patients; LR-4 probably HCC, LR-5 definitely HCC, LR-M definitely or probably malignant, not HCC specific, LR-TIV tumor in vein

*APHE* arterial phase hyperenhancement, *MVI* microvascular invasion

*Means statistical significance

### Univariable and multivariable analyses for predictve of MVI

A total of 10 characteristics with *p* < 0.1 were analyzed using univariable logistic regression, including AFP > 400 ng/mL (*p* = 0.023), LMR (*p* = 0.077), AAPR (*p* = 0.040), APRI (*p* = 0.026), ANRI (*p* = 0.017), AGLR (*p* = 0.076), NRPI (*p* = 0.044), GAR (*p* = 0.083), non-smooth margin (*p* = 0.013), and target sign on DWI (*p* = 0.055), as well as arterial phase peritumoral enhancement (*p* < 0.001). The above 7 significant variables (*p* < 0.05) were analyzed using multivariable logistic regression analysis (forward LR), which determined that higher AAPR (> 0.75; odds ratio (OR) 8.586; 95% confidence interval (CI) 1.226, 60.138; *p* = 0.030), lower ANRI (≤ 4.63; OR 0.237; 95% CI 0.061, 0.915; *p* = 0.037), non-smooth margin (OR 2.742; 95% CI 1.032, 7.284; *p* = 0.043), and arterial phase peritumoral enhancement (OR 6.167; 95% CI 2.457, 15.477; *p* < 0.001) were associated with MVI in cHCC-CCA patients (Table 3).

**Table 3:** Univariate and multivariate analyses of risk factors for the MVI of cHCC-CCA

Risk factor	Univariate analysis		Multivariate analysis	
	Odds ratio (95% CI)	*p* value	Odds ratio (95% CI)	*p* value
AFP ≥ 400, ng/mL	3.065 (1.163, 8.075)	0.023*	3.163 (0.944, 10.603)	0.062
LMR	2.109 (0.923, 4.82)	0.077		
AAPR	5.22 (1.077, 25.297)	0.040*	8.586 (1.226, 60.138)	0.030*
APRI	0.169 (0.035, 0.808)	0.026^*^		
ANRI	0.267 (0.09, 0.791)	0.017*	0.237 (0.061, 0.915)	0.037*
AGLR	0.502 (0.234, 1.074)	0.076		
NRPI	3.109 (1.031, 9.369)	0.044*		
GAR	0.525 (0.253, 1.088)	0.083		
Non-smooth margin	2.55 (1.214, 5.358)	0.013*	2.742 (1.032, 7.284)	0.043*
Target sign on DWI	0.455 (0.203, 1.018)	0.055		
Arterial phase peritumoral enhancement	8.256 (3.611, 18.877)	< 0.001*	6.167 (2.457, 15.477)	< 0.001*

*CI* confidence interval, *AFP* α-fetoprotein, *LMR* lymphocyte to monocyte ratio, *AAPR* albumin-to-alkaline phosphatase ratio, *APRI* aspartate aminotransferase-to-platelet ratio index, *ANRI* aspartate aminotransferase-to-neutrophil ratio index, *AGLR* (alkaline phosphatase + γ-glutamyltranspeptidase)-to-lymphocyte ratio, *NRPI* neutrophil-to-prealbumin ratio index, *GAR* γ-glutamyltranspeptidase-to-albumin ratio

*Indicate statistical significance

### Diagnostic performance of prediction models

**Fig. 4:**
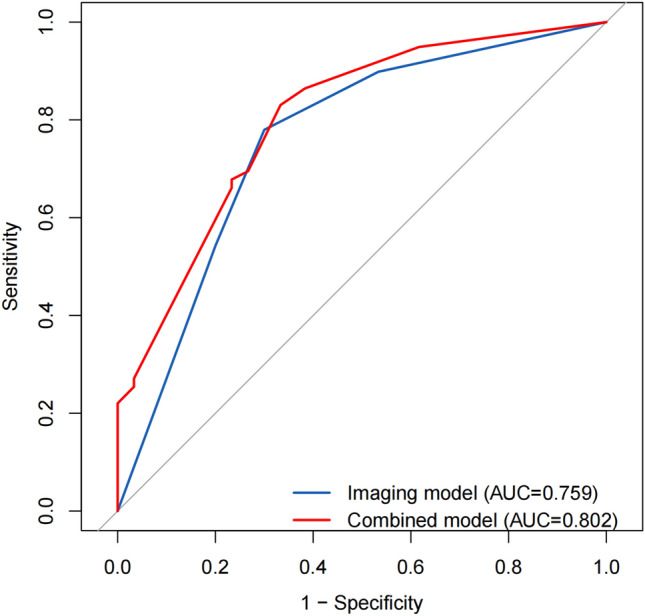
Receiver operating characteristic analysis for the combined model and imaging model, with an AUC of 0.802 and 0.759, respectively

The sensitivity, specificity, accuracy, positive predictive value (PPV), and negative predictive value (NPV) for the prediction of MVI by each significant factor and their combination are shown in Table 4. The ROC curve analysis showed that the combined model had better diagnostic performance for predicting MVI than the imaging model, with an AUC of 0.802 and 0.759, respectively (Fig. 4). When imaging features and inflammatory biomarkers were combined, the sensitivity was 83.1% (49/59), and the specificity was 66.7% (40/60).

**Table 4:** Diagnostic performance for MVI of cHCC-CCA

	AUC (95% CI)	Sensitivity	Specificity	Accuracy	PPV	NPV
AAPR	0.560 (0.456, 0.663)	15.2 (9/59)	96.7 (58/60)	56.3 (67/119)	81.8 (9/11)	53.7 (58/108)
ANRI	0.585 (0.483, 0.688)	25.4 (15/59)	91.7 (55/60)	58.9 (70/119)	75.0 (15/20)	55.6 (55/99)
Non-smooth margin	0.614 (0.512–0.715)	66.1 (39/59)	60.0 (34/60)	61.3 (73/119)	60.0 (39/65)	63.0 (34/54)
Arterial phase peritumoral enhancement	0.740 (0.648, 0.831)	78.0 (46/59)	70.0 (42/60)	73.9 (88/119)	71.9 (46/64)	76.4 (42/55)
Imaging model	0.759 (0.671, 0.847)	78.0 (46/59)	70.0 (42/60)	73.9 (88/119)	71.9 (46/64)	76.4 (42/55)
Combined model	0.802 (0.724, 0.881)	83.1 (49/59)	66.7 (40/60)	74.8 (89/119)	71.0 (49/69)	80.0 (40/50)

*Imaging model* model only using imaging data, *Combined model* combined inflammatory biomarkers with imaging features, *AUC* area under the curve, *PPV* positive predictive value, *NPV* negative predictive value

## Discussion

This study demonstrated that two MR-imaging features (non-smooth margin and arterial phase peritumoral enhancement) and two inflammatory biomarkers (AAPR and ANRI) were independent predictors of MVI in patients with cHCC-CCA. The diagnostic sensitivity and specificity of the combined model derived from MR-imaging features and inflammatory biomarkers were 83.1% and 66.7%, respectively. This combined model could help radiologists and surgeons preoperatively predict MVI in cHCC-CCA patients.

The results showed that the serum AFP level > 400 ng/mL was higher in patients with MVI than in those without MVI, but it was not an independent risk factor for MVI in cHCC-CCA. This is consistent with previous research [14, 28]. Similar to HCC and ICC, cHCC-CCA was predominantly found in males (approximately 94/119), but gender did not differ between the two groups. According to a previous report, the hepatitis B virus was prevalent in cHCC-CCA patients [29]. In this study, most of the patients (80%) with cHCC-CCA had also been infected with the hepatitis B virus.

Two inflammatory biomarkers, namely AAPR and ANRI, were significantly associated with the presence of MVI in cHCC-CCA. AAPR and ANRI incorporate routinely available laboratory parameters, including ALB and ALP, AST, and neutrophil count, respectively. Albumin is a valuable marker for determining the inflammatory response, and alkaline phosphatase hydrolase is primarily found in the liver, bile ducts, and bone [28]. In this study, a higher AAPR was confirmed to be an independent risk factor for MVI in cHCC-CCA, consistent with prior research [28]. Elevated AAPR levels may be associated with the probability of tumor invasion. Neutrophils in the primary tumor microenvironment are closely associated with the local inflammatory response and can promote tumor invasion, metastasis, and angiogenesis through the release of hepatocyte growth factor, neutrophil elastase, and matrix metalloproteins [30, 31]. AST, as a routine indicator for evaluating liver function, can reflect liver damage and is also used to assess the progression of liver disease. Ji et al. and Zheng et al. found that ANRI indicates a worse prognosis after surgery in HCC patients [32, 33]. In this study, ANRI was considered to be related to MVI in cHCC-CCA patients. Other inflammatory biomarkers, such as APRI and NPRI, were significantly linked with MVI in univariate analysis, but not in multivariate analysis. This differs from the result of Shi et al. [34], who found that APRI was one of the independent risk factors for MVI. The discrepancy may be due to tumor size and sample size limitations.

A set of MR-imaging features can confirm the presence of MVI, including a non-smooth tumor margin and arterial phase peritumoral enhancement. A recent meta-study showed that non-smooth margin is an independent risk factor for predicting MVI in HCC patients and demonstrated statistically significant imaging features [17]. Non-smooth tumor margin has been associated with more aggressive tumor growth patterns and increased MVI [35]. In addition, arterial phase peritumoral enhancement is an MR-imaging feature suggestive of MVI, consistent with the findings of Wang et al. [13]. Arterial peritumoral hyperenhancement may be due to arterial hyperperfusion compensating for decreased portal flow, which could be caused by microscopic tumor thrombi surrounding the tumor obstructing the minute portal vein branches [36, 37]. According to a recent study [38], MVI was significantly correlated with recurrence-free survival (RFS) in L4/5 and L-M after stratifying cHCC-CCA by LI-RADS 2018, but neither LR-4/5 nor LR-M was associated with MVI in the present study. The effect of the LI-RADs category on predicting MVI needs to be further evaluated in future studies.

The strength of this study lies in the combination of MR-imaging features and inflammatory biomarkers, both derived from routine clinical practice, to preoperatively predict the presence of MVI in patients with cHCC-CCA. Our combined model, which integrates two MR-imaging features and two inflammatory biomarkers, achieved an AUC of 0.802.

However, this study also had several limitations. First, as a single-center retrospective study limited to our medical institution, there may be potential selection bias affecting the reproducibility and comparability of the results. Second, imaging features on the hepatobiliary stage were not included in this study, despite the widely recognized value of the hepatobiliary stage in predicting MVI in HCC patients. Combining gadoxetic acid-enhanced MRI would provide more comprehensive and valuable information for predicting MVI in cHCC-CCA patients. Third, the low incidence of cHCC-CCA and the small number of patients make it difficult to conduct further graded studies of MVI based on the number of invading vessels. Fourth, survival data were not evaluated in this study due to the high rate of patient loss to follow-up. In the future, additional samples and multicenter study will be collected to further refine and validate our findings.

## Conclusions

In conclusion, this study retrospectively evaluated the clinical applicability and value of MR-imaging features and inflammatory biomarkers in predicting MVI in cHCC-CCA patients. Four independent predictors, including two MR-imaging features and two inflammatory biomarkers, were used to construct a preoperative risk model for MVI. As a result, the combined model allowed for accurate preoperative prediction of MVI and may aid in tailoring personalized treatment decisions.

### Supplementary Information

Below is the link to the electronic supplementary material.

Supplementary file1 (DOCX 12 kb)

Supplementary file2 (DOCX 14 kb)

## Data Availability

The data that support the findings of this study are available from the corresponding author upon reasonable request**.**

## Abbreviations

ALB: Albumin
ALT: Alanine aminotransferase
AFP: Alpha-fetoprotein
APHE: Arterial phase hyperenhancement
AST: Aspartate aminotransaminase
AUC: Area under curve
CA19-9: Carbohydrate antigen 19-9
CEA: Carcinoembryonic antigen
cHCC-CCA: Combined hepatocellularcholangiocarcinoma
CI: Confidence interval
DB: Direct bilirubin
GGT: γ-Glutamyltranspeptidase
HBV: Hepatitis B virus
HCC: Hepatocellular carcinoma
ICC: Intrahepatic cholangiocarcinoma
LI-RADS: Liver Imaging Reporting and Data System
MVI: Microvascular invasion
MRI: Magnetic resonance imaging
NPV: Negative predictive value
OR: Odds ratio
PIVKA-II: Protein induced by vitamin K absence or antagonist-II
PLC: Primary liver cancer
PPV: Positive predictive value
pro-ALB: Pro-albumin
TB: Total bilirubin
