# Supplementary material for: Preoperative evaluation of MRI features and inflammatory biomarkers in predicting microvascular invasion of combined hepatocellular cholangiocarcinoma
Source: Abdom Radiol (NY). 2023 Dec 19;49(3):710–21. doi: 10.1007/s00261-023-04130-6 (PMC10909765; doi:10.1007/s00261-023-04130-6)
Supplement: Supplementary file 1 — Supplementary file1 (DOCX 12 kb) [file 261_2023_4130_MOESM1_ESM.docx]

**Supplementary Table S1.** **Detailed sequences and parameters**

| **Sequences** | **TR/TE (msec)** | **FOV (mm)** | **Flip angle** | **Thickness (mm)** | **Matrix** |
| --- | --- | --- | --- | --- | --- |
| T1WI | 190/4.3(2) | 420 × 420 | 80 | 6 | 256 × 160 |
| T2WI | 6667/85 | 420 × 420 | 160 | 6 | 320 × 224 |
| DCE | 3.7/1.7 | 420 × 420 | 15 | 2.5 | 256 × 192 |
| 2D MRCP | 4000/847 | 320 × 320 | 160 | 50 | 320 × 256 |

*DCE*, dynamic contrast-enhanced
