# Supplementary material for: Preoperative evaluation of MRI features and inflammatory biomarkers in predicting microvascular invasion of combined hepatocellular cholangiocarcinoma
Source: Abdom Radiol (NY). 2023 Dec 19;49(3):710–21. doi: 10.1007/s00261-023-04130-6 (PMC10909765; doi:10.1007/s00261-023-04130-6)
Supplement: Supplementary file 2 — Supplementary file2 (DOCX 14 kb) [file 261_2023_4130_MOESM2_ESM.docx]

**Supplementary Table S2.** Inter-reader agreement of MR imaging features and LI-RADS category

|  | Cohen's kappa |
| --- | --- |
| Tumor Margin | 0.800 (0.694, 0.906) |
| Intratumoral necrosis | 0.637 (0.500, 0.778) |
| Fat deposition | 0.760 (0.534, 0.985) |
| Intratumoral hemorrhage | 0.608 (0.433, 0.782) |
| Non-rim APHE | 0.848 (0.754, 0.942) |
| Arterial phase peritumoral enhancement | 0.865 (0.775, 0.955) |
| Washout | 0.799 (0.691, 0.907) |
| Enhancing capsule | 0.848 (0.746, 0.950) |
| Delayed central enhancement | 0.848 (0.746, 0.950) |
| Nodule in nodule | 0.785 (0.642, 0.928) |
| Mosaic architecture | 0.781 (0.661, 0.901) |
| Peritumoral bile duct dilatation | 0.827 (0.662, 0.992) |
| Hepatic capsule retraction | 0.879 (0.785, 0.973) |
| Target sign on DWI | 0.807 (0.693, 0.921) |
| LI-RADS category | 0.756 (0.644 ,0.868) |

Data in parentheses are 95% confidence intervals.

*LI-RADS*, Liver Imaging Reporting and Data System

*Non-rim APHE*, non-rim arterial phase hyperenhancement
